# ^1^H, ^13^C, ^15^N and ^31^P chemical shift assignment for stem-loop 4 from the 5′-UTR of SARS-CoV-2

**DOI:** 10.1007/s12104-021-10026-7

**Published:** 2021-04-29

**Authors:** Jennifer Vögele, Jan-Peter Ferner, Nadide Altincekic, Jasleen Kaur Bains, Betül Ceylan, Boris Fürtig, J. Tassilo Grün, Martin Hengesbach, Katharina F. Hohmann, Daniel Hymon, Bozana Knezic, Frank Löhr, Stephen A. Peter, Dennis Pyper, Nusrat S. Qureshi, Christian Richter, Andreas Schlundt, Harald Schwalbe, Elke Stirnal, Alexey Sudakov, Anna Wacker, Julia E. Weigand, Julia Wirmer-Bartoschek, Jens Wöhnert, Elke Duchardt-Ferner

**Affiliations:** 1 Institute for Molecular Biosciences, Goethe-University Frankfurt, Max-von-Laue-Str. 9, 60438 Frankfurt/M., Germany; 2 Center for Biomolecular Magnetic Resonance (BMRZ), Goethe-University Frankfurt, Max-von-Laue-Str. 7, 60438 Frankfurt/M., Germany; 3 Institute for Organic Chemistry and Chemical Biology, Goethe-University Frankfurt, Max-von-Laue-Str. 7, 60438 Frankfurt/M., Germany; 4 Institute for Biophysical Chemistry, Goethe-University Frankfurt, Max-von-Laue-Str. 9, 60438 Frankfurt/M., Germany; 5 Department of Biology, Technical University of Darmstadt, Schnittspahnstr. 10, 64287 Darmstadt, Germany; 6 Present Address: EMBL Heidelberg, Meyerhofstraße 1, 69117 Heidelberg, Germany

**Keywords:** SARS-CoV-2, RNA genome, 5′-UTR, 5_SL4, Solution NMR spectroscopy, COVID19-NMR

## Abstract

The SARS-CoV-2 virus is the cause of the respiratory disease COVID-19. As of today, therapeutic interventions in severe COVID-19 cases are still not available as no effective therapeutics have been developed so far. Despite the ongoing development of a number of effective vaccines, therapeutics to fight the disease once it has been contracted will still be required. Promising targets for the development of antiviral agents against SARS-CoV-2 can be found in the viral RNA genome. The 5′- and 3′-genomic ends of the 30 kb SCoV-2 genome are highly conserved among Betacoronaviruses and contain structured RNA elements involved in the translation and replication of the viral genome. The 40 nucleotides (nt) long highly conserved stem-loop 4 (5_SL4) is located within the 5′-untranslated region (5′-UTR) important for viral replication. 5_SL4 features an extended stem structure disrupted by several pyrimidine mismatches and is capped by a pentaloop. Here, we report extensive ^1^H, ^13^C, ^15^N and ^31^P resonance assignments of 5_SL4 as the basis for in-depth structural and ligand screening studies by solution NMR spectroscopy.

**Supplementary Information:**

The online version contains supplementary material available at 10.1007/s12104-021-10026-7.

## Biological context

SARS-CoV-2 is a human Betacoronavirus which causes the severe acute respiratory syndrome COVID-19. The virus contains a large single-stranded ( +) RNA genome with a length of approximately 30,000 nt. Besides the coding regions for the viral proteins, the genome also includes extended, highly structured and conserved 5′- and 3′-untranslated regions (UTRs) with important functional roles in genome replication, transcription of subgenomic (sg) mRNAs and the balanced translation of viral proteins. So far, efforts aiming at the development of new antiviral agents against SARS-CoV-2 have been largely restricted to studies of the viral proteins, leaving the potentially vast reservoir of putative drug-targets to be found in the structured, conserved and functional genomic RNA elements essentially untapped. Triggered by the COVID-19 pandemic, the Covid19-NMR initiative (https://covid19-NMR.de) has united structural biologists and RNA biologists around the globe in a concerted initiative to make these viral RNA elements amenable as therapeutic targets as well as to pilot structure-guided drug screening efforts against these RNA targets. At the heart of this effort is the conviction that drug development can profit from and be efficiently guided by high resolution structural data. As a starting point of the initiative, the individual structured elements of the SARS-CoV-2 genome were therefore subjected to high resolution structure determination by NMR spectroscopy in a ‘divide-and-conquer’ approach.

**Fig. 1:**
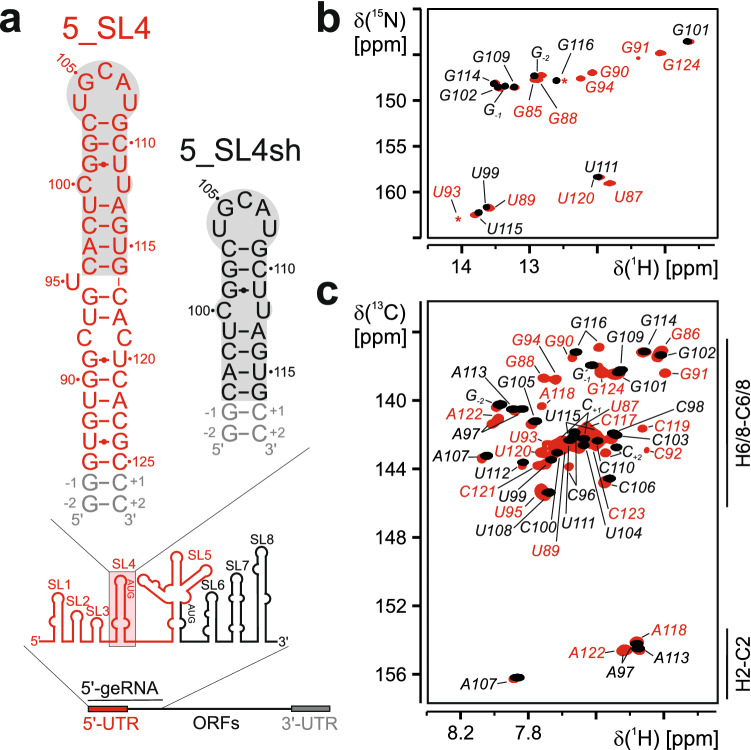
**a** Sequence and secondary structure of the 44 nt 5_SL4 (top, left) and the smaller construct 5_SL4sh (top, right) and genomic context of 5_SL4. Scheme of the SARS-CoV2 genome (bottom) and overview of cis-acting RNA elements of the 5′ genomic end (middle). **b** Overlay of the imino ^1^H, ^15^N-BEST-TROSY spectra of 5_SL4 (red) and 5_SL4sh (black). Assignments are given. The asterisks mark signals that are visible at a lower contour threshold. **c** Overlay of the aromatic region of ^1^H,^13^C-HSQC spectra of 5_SL4 (red) and 5_SL4sh (black). Assignments are given. Those that only belong to the full-length construct are highlighted in red

The 5′- region (Fig. 1a) of the SARS-CoV-2 genome consists of eight stem-loop (SL) structures. Stem-loops 5_SL1 to 5_SL5 are located in the 5′-untranslated region (5′-UTR). While the sequences of the individual structural elements vary between different coronaviruses, their ubiquitous presence and highly conserved secondary structures suggest that these elements are critically important for viral viability and pathogenesis (Madhugiri et al. 2016). Stem-loop 4 of the 5′-UTR (5_SL4, nt 86–125), a 40 nt predicted hairpin capped by a pentaloop, is structurally conserved among the members of the Betacoronavirus family. Interestingly, 5_SL4 carries an upstream open reading frame (uORF) with its AUG start codon as integral part of the stem. This uORF is conserved within the Betacoronaviruses. Its function, however, is still under debate. On the one hand, genetic pressure to preserve the uORF has been observed. On the other hand, mutations manipulating the uORF yet retaining the 5_SL4 structure were still viable (Wu et al. 2014). We have recently established the secondary structure of 5_SL4 based on initial ^1^H and ^15^N NMR resonance assignments (Wacker et al. 2020). As a further step to guide structure-based studies of 5_SL4 amenable we provide here the almost complete ^1^H, ^13^C,^15^N and ^31^P NMR chemical shift assignment.

## Methods and experiments

In order to adapt 5_SL4 for enzymatic synthesis, the 40 nt sequence (residues 86–125 of the SARS_COV2 genome) was extended by two guanine residues at the 5′- and two cytidine residues at the 3′-end yielding the 44 nt sequence 5′-GG**GUGUGGCUGUCACUCGGCUGCAUGCUUAGUGCACUCACGC**CC-3′ (5_SL4) with the wt-sequence shown in bold letters. In addition, a shorter construct comprising only the apical residues 96–116 again flanked by two G-C base pairs was synthesized (5_SL4sh, 25 nt, sequence 5′-GG**CACUCGGCUGCAUGCUUAGUG**CC-3′). Of 5_SL4, a uniformly ^15^N- and selectively A/C- and G/U-^13^C/^15^N-labeled samples and of 5_SL4sh a uniformly ^13^C/^15^N-labeled sample were prepared as described in detail previously (Wacker et al. 2020). The final RNA concentrations in all NMR samples varied between 0.35 and 0.79 mM in 25 mM potassium phosphate buffer, pH 6.2, with 50 mM potassium chloride and either 5% or 100% (v/v) D_2_O.

NMR measurements were carried out at the Center for Biomolecular Magnetic Resonance (BMRZ) on 600, 800, 900 and 950 MHz Bruker Avance NMR-spectrometers equipped with 5-mm cryogenic triple resonance TCI-N probe heads, a 700 MHz spectrometer equipped with a quadruple resonance QCI-P probe and an 800 MHz spectrometer equipped with a ^13^C-optimized TXO cryogenic probe (800 MHz^TXO^). ^1^H chemical shifts were referenced directly to an external DSS standard, and ^13^C, ^15^N, ^31^P and chemical shifts were indirectly referenced from the ^1^H chemical shift as described earlier (Maurer and Kalbitzer 1996; Wishart et al. 1995). All NMR experiments conducted for the resonance assignment of 5_SL4 and 5_SL4sh are summarized in Supplementary Table 1. If not indicated otherwise, experiments were performed at 25 °C. NMR data were processed using TOPSPIN 4.0.6 software (Bruker, BioSpin, Germany) and analyzed using CARA (Keller 2004).

## Extent of assignments and data deposition

5_SL4 is a 44 nt long predicted stem-loop capped by an apical loop of five nucleotides (Fig. 1a). Given the rather large size of this RNA together with its expected rod-like extended shape and high content of canonical base-pairs, unfavorable relaxation behavior is expected to combine with limited resonance dispersion to interfere with a complete resonance assignment. We therefore also investigated a smaller construct containing only the apical stem loop and the predicted C100-U112 mismatch (5_SL4sh, Fig. 1a) to allow for an unambiguous sequential assignment of the apical loop as well as the acquisition of the chemical shifts of the mismatch residues as a prerequisite for the subsequent structure determination. For transcriptional reasons we added two terminal G-C base pairs at the end of the stem in both constructs.

The comparison of the imino ^1^H,^15^N-TROSY spectra of the full-length construct and 5_SL4sh indicates the complete preservation of the RNA structure in the truncated variant (Fig. 1b). This is also confirmed by comparing the aromatic region of ^1^H,^13^C-HSQC spectra recorded for both constructs (Fig. 1c). Only residues at the bottom of the 5_SL4sh stem display minor shift changes compared to the full length construct as expected.

We have previously reported an initial resonance assignment of 5_SL4 comprising the imino and amino groups and extending to the aromatic and H1′ protons (Wacker et al. 2020). On that basis, we followed essentially the classical assignment pathway using the NMR experiments listed in Supplementary Table S1. For 5_SL4sh, the assignment of the imino- and amino-group resonances could be readily transferred from the full length 5_SL4 assignment in ^15^ N HSQC spectra optimized for the imino- and the amino group region, respectively. Based on the previous assignment of the aromatic proton spins, ^1^H,^13^C-sfHMQC and ^1^H,^13^C-HSQC spectra for the aromatic region served to assign 100% of the H2-C2 and H6/8-C6/8 resonances. 3D ^13^C-NOESY-HSQC spectra confirmed this assignment. All of the adenine N1 and N3 and the purine N7 and N9 resonances were assigned in the lr-^1^H,^15^N-HSQC. Nine out of 14 guanine N3 resonances were observed in the HNN-COSY spectrum of 5_SL4. With the exception of C100, all pyrimidine N3 resonances were assigned in the H5(C5C4)N3 spectrum for 5_SL4sh. Out of the additional 15 pyrimidine residues in 5_SL4, N3 signals for 10 were assigned in ^1^H,^15^N HSQC and HNN-COSY spectra. All pyrimidine N1 resonances in 5_SL4 were assigned using the carbon detected 3D (H6/8)C6/8N1/9C1′ experiments, which also served link the aromatic carbons to the C1′ resonances for all residues. Using ^1^H,^13^C-HSQC and 3D ^13^C-NOESY-HSQC spectra for the aliphatic region 100% of the H1′–C1′ and the H5–C5 resonances could be assigned. The remaining ribose carbon resonances were assigned using 3D (H)CCH-TOCSY spectra. The complete assignment of the ribose protons was then achieved by 3D HC(C)H-COSY, -TOCSY experiments for 5_SL4 and using 3D ^13^C-NOESY-HSQC spectra for 5_SL4sh.

### Assignment and ribose conformation of the apical loop

**Fig. 2:**
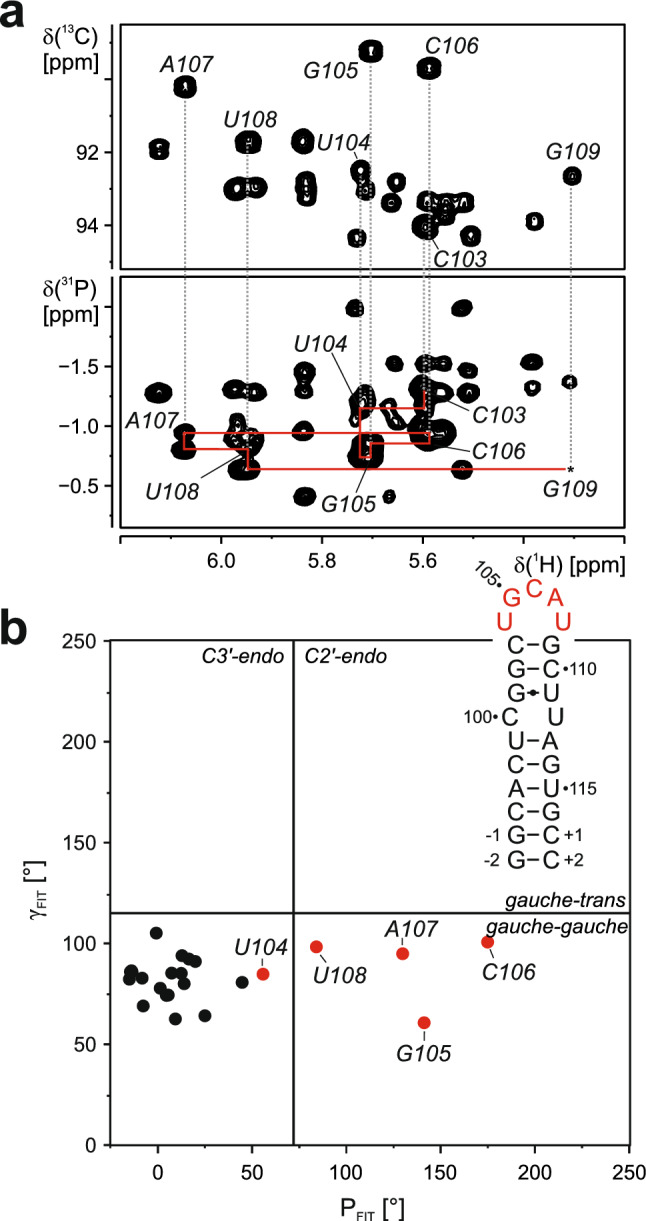
Assignment and ribose conformation of the 5_SL4 apical loop. **a** H1′C1′ region of a ^1^H, ^13^C HSQC spectrum (top) and H(C)P-CCH-TOCSY spectrum (bottom) recorded for the 5_SL4sh RNA. Sequential correlations between the residues in the loop are shown as red lines. **b** Canonical coordinates for all but the 3′-terminal residue of 5_SL4sh The secondary structure of 5_SL4sh is displayed. Residues of the apical loop are highlighted in red and assignments are given

5_SL4 is capped by an apical loop comprising nucleotides 104 to 108 with the sequence 5ʹ-U_104_GCAU_108_-3ʹ. The sequential assignment of the loop residues solely from NOE contacts was ambiguous. Therefore, we recorded a H(C)P-CCH-TOCSY spectrum for the 5_SL4sh RNA establishing the sequential ribose spin system assignment for this part (Fig. 2a). Furthermore, all 27 ^31^P resonances for 5_SL4sh were assigned using this spectrum together with the 1D ^31^P spectrum, which also served to assign the characteristic C25 cyclic phosphate and the α, β and γ phosphate resonance of the 5′-terminal G residue.

Extensive assignment of the ribose carbon spins allows for the extraction of canonical coordinates yielding information about the sugar pucker mode (Cherepanov et al. 2010; Ebrahimi et al. 2001) (Fig. 2b). A C2′-endo conformation was found for the apical loop residues G105 to U108, while U104 from the loop and all remaining residues in 5_SL4sh adopt a C3′-endo conformation.

### Base pairing interactions

**Fig. 3:**
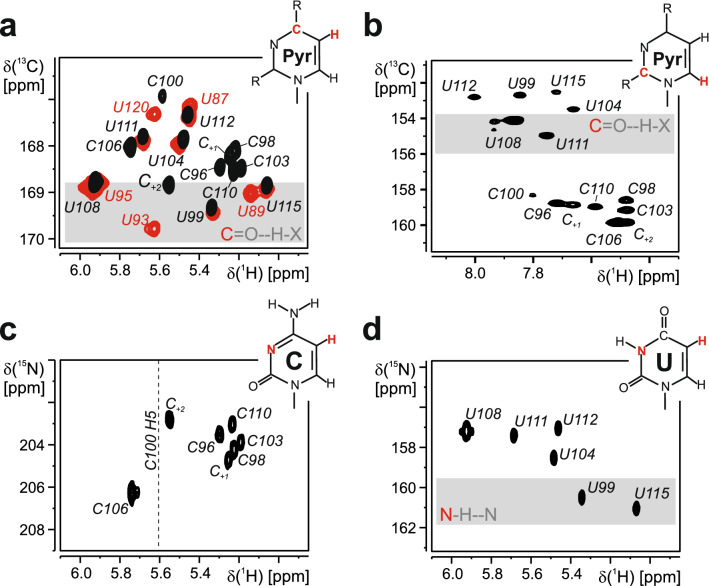
Carbon and nitrogen chemical shifts of quaternary carbon and the nitrogen spins in the pyrimidine residues. **a** 2D H5(C5)C4 spectrum recorded for 5_SL4 (red) and 5_SL5sh (black). Assignments are given. The chemical shift region of the carbon atoms of carbonyl groups involved in hydrogen bonds is highlighted with a gray bar. The chemical structure of pyrimidine bases is displayed. The H5 and C4 atoms are highlighted in red. **b** H6(C6N1)C2 spectra of 5_SL4sh. Resonance assignments are given. The chemical structure of a pyrimidine base is shown with H6 and C2 highlighted in red. **c, d** 2D H5(C5C4)N3 spectra recorded for the cytidines (**c**) and the uridines (**d**) of 5_SL4sh. The chemical structures of cytidine (C) and uridine (U) bases are displayed. The H5 and N3 atoms are highlighted in red. The chemical shift of the H5 of C100 is shown as a dashed line. The chemical shift region of nitrogen atoms of imino groups that are involved in hydrogen bonds is marked with a gray bar

In general, the secondary structure of 5_SL4 has been established previously by combination of 2D imino NOESY data with HNN-COSY spectra (Wacker et al. 2020). HNN-COSY spectra verified the identity of the canonical Watson–Crick A–U and G–C base pairs in the stem-loop structure, while of the three potential G–U base pairs, only U87–G124 and G101–U111 could be readily identified by virtue of their strong intra-base-pair imino-imino NOEs in the NOESY spectrum (Wacker et al. 2020). For the remaining G–U base pair (G91–U120), only a very weak guanine imino resonance was identified. For U87 and U111, which possess detectable imino resonances, C2 and C4 shifts could be obtained in an 2D H(N)CO spectrum (data not shown). Downfield C2 and upfield C4 shifts for both U87 and U111 point to a classical wobble-arrangement for the respective G–U base pairs, with the G imino group hydrogen bonded to the U C2. For the G91–U120 base pair, the uridine is missing an imino resonance. In order to investigate the C4 chemical shift of this residue even in the absence of a stable imino resonance, a H5(C5)C4 spectrum was recorded (Fig. 3a). The C4 of U120 has a resonance frequency very similar to both U87 and U111, suggesting a similar Wobble base pair geometry for this G–U base pair.

The carbon and nitrogen chemical shifts for carbon and nitrogen nuclei not directly bound to protons in the C100–U112 mismatch were investigated using 5_SL4sh. Since the imino proton of U112 is not observable the information about this potential base pair has been very limited. The investigation of the smaller construct enabled the additional assignment of quaternary carbon spins in the pyrimidine nucleobases which can be predictive for base functional group hydrogen bonding patterns (Ohlenschläger et al. 2004). Using a 2D H5(C5)C4 spectra, 100% of the pyrimidine C4 carbon atoms were assigned (Fig. 3a). Compared to the C4 carbon chemical shift of U115 and U99 whose C4 carbonyl groups are hydrogen bonded in A–U base pairs, the C4 of U112 is shifted upfield, indicating no involvement of the C4 carbonyl group in hydrogen bonding interactions. A 2D H6(C6N1)C2 experiment was used to identify the C2 resonances of all C and U residues of 5_SL4sh (Fig. 3b). The resonance of U112 is shifted upfield compared to the of U111 in the G–U base pair, for which the C2 carbonyl group is hydrogen bonded to the G101 imino group. Taken together carbonyl chemical shifts for U112 suggests that neither the C2 nor the C4 of this residue is involved in stable hydrogen bond interactions. Using 2D H5(C5C4)N3 spectra, seven out of eight cytidine (Fig. 3c) and 100% of the uridine N3 nitrogen resonances (Fig. 3d) were assigned. The N3 resonance of U112 is shifted upfield compared to the N3 of U99 and U115 that are involved in N–H···N type hydrogen bonds suggesting that the U112 imino group is not involved in such an interaction with C100. The N3 nitrogen of C100 is not detectable. Hence, the structure and putative dynamics of the C100–U112 mismatch are still subject for further investigations.

### Data deposition

For 5_SL4, we updated the BMRB deposition with code 50347. For 5_SL4sh a new BMRB entry (50760) was created.

## Supplementary Information

Below is the link to the electronic supplementary material.

Supplementary file1 (DOCX 62 kb)
