# Supplementary material for: 1H, 13C, 15N and 31P chemical shift assignment for stem-loop 4 from the 5′-UTR of SARS-CoV-2
Source: Biomol NMR Assign. 2021 Apr 29;15(2):335–40. doi: 10.1007/s12104-021-10026-7 (PMC8083917; doi:10.1007/s12104-021-10026-7)
Supplement: Supplementary file 1 — Supplementary file1 (DOCX 62 kb) [file 12104_2021_10026_MOESM1_ESM.docx]

**Supplementary Information: ^1^H, ^13^C, ^15^N and ^31^P chemical shift assignment for stem-loop 4 from the 5'-UTR of SARS-CoV-2**

**Supplementary Table S1:** List of NMR experiments conducted for the resonance assignment of 5_SL4. Experimental parameters are given. ns = number of scans, sw = spectral width, aq = acquisition time, o1-o4 = carrier frequencies on channels 1-4, rel. delay = relaxation delay. The different samples are indicated in bold according to their isotope labeling scheme. Parameters for 5_SL4sh are highlighted in italic. For 5_SL4sh a uniformly ^13^C^15^N labeled sample was used.

| **NMR experiment** | **Experimental parameters** |
| --- | --- |
| **^1^H,^15^N-BEST-TROSY**  imino group region  (Favier and Brutscher 2011; Solyom et al. 2013)(Solyom et al. 2013; Favier und Brutscher 2011) | *800 MHz^TXO^, ns: 16, sw(f2): 21 ppm, sw(f1): 25 ppm, aq(f2): 60 ms, aq(f1): 128 ms, o1(^1^H): 4.7 ppm, o2(^13^C): 101 ppm, o3(^15^N): 153 ppm, rel. delay: 0.3 s, time: 1 h (10 °C)* |
| **^1^H,^15^N-HSQC**  amino group region  (Mori et al. 1995) | *800 MHz^TXO^, ns: 48, sw(f2): 20 ppm, sw(f1): 35 ppm, aq(f2): 32 ms, aq(f1): 45 ms, o1(^1^H): 4.7 ppm, o2(^13^C): 101 ppm, o3(^15^N): 88 ppm, rel. delay: 1.0 s, time: 4 h (10 °C)* |
| **^1^H, ^15^N-CPMG-NOESY**  (Mueller et al. 1995) | *800 MHz, ns: 288, sw(f2): 22 ppm, sw(f1): 103 ppm, aq(f2): 59 ms, aq(f1): 12 ms, o1(^1^H): 4.7 ppm, o2(^13^C): 101 ppm, o3(^15^N): 116 ppm, rel. delay: 1.0 s, time: 19 h (10 °C)* |
| **^1^H,^13^C-sfHMQC**  aromatic region  (Schanda and Brutscher 2005) | **^15^N**: 800 MHz^TXO^, ns: 896, sw(f2): 10 ppm, sw(f1): 25 ppm, aq(f2): 63 ms, aq(f1): 15 ms, o1(^1^H): 4.7 ppm, o2(^13^C): 143 ppm, o3(^15^N): 190 ppm, rel. delay: 0.3 s, time: 14 h |
| **^1^H,^13^C-HSQC** | **Optimized for aromatic C-H moieties:**  INEPT transfer time 2.7 ms (^1^J_CH_ 185 Hz), off-resonant Q3 shaped pulse for C5 decoupling at 95 ppm with 25 ppm bandwidth (900 µs at 600 MHz, 680 µs at 800 MHz).  **G/U-^13^C/^15^N**: 600 MHz, ns: 8, sw(f2): 8.3 ppm, sw(f1): 12 ppm, aq(f2): 102 ms, aq(f1): 141 ms, o1(^1^H): 4.7 ppm, o2(^13^C): 136.5 ppm, o3(^15^N): 153 ppm, rel. delay: 1.0 s, time: 1 h  **A/C-^13^C/^15^N**: 800 MHz^TXO^, ns: 8, sw(f2): 8.6 ppm, sw(f1): 25 ppm, aq(f2): 75 ms, aq(f1): 38 ms, o1(^1^H): 4.7 ppm, o2(^13^C): 143 ppm, o3(^15^N): 153 ppm, rel. delay: 1.0 s, time: 1 h  *800 MHz^TXO^, ns: 8, sw(f2): 8.6 ppm, sw(f1): 25 ppm, aq(f2): 75 ms, aq(f1): 38 ms, o1(^1^H): 4.7 ppm, o2(^13^C): 143 ppm, o3(^15^N): 153 ppm, rel. delay: 1.0 s, time: 1 h*  **Optimized for aliphatic C-H moieties:**  INEPT transfer time 3.2 ms (^1^J_CH_ 158 Hz).  **G/U-^13^C/^15^N**: 950 MHz, ns: 4, sw(f2): 10 ppm, sw(f1): 45 ppm, aq(f2): 54 ms, aq(f1): 12 ms, o1(^1^H): 4.7 ppm, o2(^13^C): 82 ppm, o3(^15^N): 1 ppm, rel. delay: 1.0 s, time: 20 min  **A/C-^13^C/^15^N**: 600 MHz, ns: 4, sw(f2): 10 ppm, sw(f1): 40 ppm, aq(f2): 85 ms, aq(f1): 21 ms, o1(^1^H): 4.7 ppm, o2(^13^C): 79 ppm, o3(^15^N): 1 ppm, rel. delay: 1.2 s, time: 20 min  *900 MHz, ns: 4, sw(f2): 10 ppm, sw(f1): 44 ppm, aq(f2): 56 ms, aq(f1): 13 ms, o1(^1^H): 4.7 ppm, o2(^13^C): 82 ppm, o3(^15^N): 1 ppm, rel. delay: 1.0 s, time:* 20 min |
| **3D ^13^C-detected (H)CNC**  C6/8-to-C1’  Modified from  (Fiala et al. 1998) | C6/8-N1/9 transfer time 30 ms, C-H transfer time 2.9 ms (C1’) and 2.6 ms (C6/8)  **G/U-^13^C/^15^N**: 800 MHz^TXO^, ns: 16, sw(f3): 24.5 ppm, sw(f2): 34.7 ppm, sw(f1): 12.0 ppm, aq(f3): 68 ms, aq(f2): 57 ms, aq(f1): 22 ms, o1(^13^C): 9 0 ppm, o2(^1^H): 7.6 ppm, o3(^15^N): 157 ppm, rel. delay: 0.5 s, time: 2 d  **A/C-^13^C/^15^N**: 800 MHz^TXO^, ns: 24, sw(f3): 24.5 ppm, sw(f2): 34.7 ppm, sw(f1): 15.1 ppm, aq(f3): 67 ms, aq(f2): 5.6 ms, aq(f1): 20 ms, o1(^13^C): 90 ppm, o2(^1^H): 7.6 ppm, o3(^15^N): 157 ppm, rel. delay: 0.5 s, time: 2 d 12 h |
| **3D (H)CCH TOCSY**  C1’-to-C2’  (Kay et al. 1993; Richter et al. 2010) | CC-TOCSY mixing time 6 ms  **A/C-^13^C/^15^N**: 800 MHz, ns: 8, sw(f3,^1^H): 8.4 ppm, sw(f2,^13^C): 9.5 ppm, sw(f1,^13^C): 35.5 ppm, aq(f3): 76 ms, aq(f2): 13 ms, aq(f1): 9 ms, o1(^1^H): 4.7 ppm, o2(^13^C): 76.5 ppm, o3(^15^N): 153 ppm, rel. delay: 1.0 s, time: 16 h |
| **3D (H)CCH TOCSY**  C1’-to-C5’  (Kay et al. 1993; Richter et al. 2010) | CC-TOCSY mixing time 18 ms.  **G/U-^13^C/^15^N**: 800 MHz, ns: 8, sw(f3,^1^H): 8.6 ppm, sw(f2,^13^C): 9.5 ppm, sw(f1,^13^C): 35.5 ppm, aq(f3): 75 ms, aq(f2): 17 ms, aq(f1): 11 ms, o1(^1^H): 4.7 ppm, o2(^13^C): 76.5 ppm, o3(^15^N): 153 ppm, rel. delay: 1.0 s, time: 3 h  **A/C-^13^C/^15^N**: 800 MHz, ns: 8, sw(f3,^1^H): 8.6 ppm, sw(f2,^13^C): 9.5 ppm, sw(f1,^13^C): 35.5 ppm, aq(f3): 75 ms, aq(f2): 13 ms, aq(f1): 11 ms, o1(^1^H): 4.7 ppm, o2(^13^C): 76.5 ppm, o3(^15^N): 153 ppm, rel. delay: 1.0 s, time: 20 h |
| **3D HC(C)H TOCSY**  (Kay et al. 1993; Pardi and Nikonowicz 1992) | CC-TOCSY mixing time: 9.2 ms  **G/U-^13^C/^15^N**: 600 MHz, ns: 4, sw(f3,^1^H): 10 ppm, sw(f2,^13^C): 42 ppm, sw(f1,^1^H): 5.5 ppm, aq(f3): 170 ms, aq(f2): 88 ms, aq(f1): 42 ms, o1(^1^H): 4.7 ppm, o2(^13^C): 80 ppm, o3(^15^N): 151 ppm, rel. delay: 1.0 s, time: 9 h |
| **3D HC(C)H COSY**  (Kay et al. 1993; Pardi and Nikonowicz 1992) | **G/U-^13^C/^15^N**: 600 MHz, ns: 4, sw(f3,^1^H): 10 ppm, sw(f2,^13^C): 42 ppm, sw(f1,^1^H): 5.5 ppm, aq(f3): 170 ms, aq(f2): 7 ms, aq(f1): 39 ms, o1(^1^H): 4.7 ppm, o2(^13^C): 80 ppm, o3(^15^N): 151 ppm, rel. delay: 1.0 s, time: 9 h  **A/C-^13^C/^15^N**: 600 MHz, ns: 8, sw(f3,^1^H): 10 ppm, sw(f2,^13^C): 38 ppm, sw(f1,^1^H): 3.2 ppm, aq(f3): 85 ms, aq(f2): 11 ms, aq(f1): 34 ms, o1(^1^H): 4.7 ppm, o2(^13^C): 79 ppm, o3(^15^N): 119 ppm, rel. delay: 1.0 s, time: 20 h |
| **3D ^13^C-NOESY-HSQC**  (Piotto et al. 1992; Sklenar et al. 1993) | **Optimized for aliphatic C-H moieties:**  NOE mixing time 200 ms, HSQC transfer time 3.2 ms (^1^J_CH_ 158 Hz).  **G/U-^13^C/^15^N**: 950 MHz, ns: 8, sw(f3,^1^H): 10 ppm, sw(f2,^13^C): 44 ppm, sw(f1,^1^H): 9.5 ppm, aq(f3): 54 ms, aq(f2): 3.8 ms, aq(f1): 14 ms, o1(^1^H): 4.7 ppm, o2(^13^C): 82.5 ppm, o3(^15^N): 115 ppm, rel. delay: 1.3 s, time: 2d 22 h  **A/C-^13^C/^15^N**: 800 MHz^TXO^, ns: 8, sw(f3,^1^H): 10 ppm, sw(f2,^13^C): 41 ppm, sw(f1,^1^H): 10 ppm, aq(f3): 64 ms, aq(f2): 5 ms, aq(f1): 17 ms, o1(^1^H): 4.7 ppm, o2(^13^C): 80 ppm, o3(^15^N): 153 ppm, rel. delay: 1.2 s, time: 3 d 8 h  *900 MHz, ns: 8, sw(f3,^1^H): 10.1 ppm, sw(f2,^13^C): 44 ppm, sw(f1,^1^H): 10.1 ppm, aq(f3): 56 ms, aq(f2): 3.9 ms, aq(f1): 16 ms, o1(^1^H): 4.7 ppm, o2(^13^C): 82 ppm, o3(^15^N): 190 ppm, rel. delay: 1.1 s, time: 2 d 18 h*  **Optimized for aromatic C-H moieties:**  NOE mixing time 200 ms, HSQC transfer time 2.8 ms (^1^J_CH_ 180 Hz).  **G/U-^13^C/^15^N**: 600 MHz, ns: 16, sw(f3,^1^H): 8.3 ppm, sw(f2,^13^C): 12.0 ppm, sw(f1,^1^H): 11.1 ppm, aq(f3): 102 ms, aq(f2): 20 ms, aq(f1): 15 ms, o1(^1^H): 4.7 ppm, o2(^13^C): 136.5 ppm, o3(^15^N): 153 ppm, rel. delay: 1.0 s, time: 3 d 12 h  **A/C-^13^C/^15^N**: 800 MHz^TXO^, ns: 16, sw(f3, ^1^H): 8.8 ppm, sw(f2,^13^C): 17.8 ppm, sw(f1, ^1^H): 11,1 ppm, aq (f3): 73 ms, aq(f2): 90 ms, aq(f1): 118 ms, o1(^1^H): 4.7 ppm, o2(^13^C): 144.3 ppm, o13(^1^H): 8.5 ppm, rel delay: 1.0 s, time: 2 d 18 h  *800 MHz, ns: 8, sw(f3,^1^H): 8.8 ppm, sw(f2,^13^C): 21 ppm, sw(f1,^1^H): 13  ppm, aq(f3): 73 ms, aq(f2): 0.06 ms, aq(f1): 21 ms, o1(^1^H): 4.7 ppm, o2(^13^C): 77 ppm, o3(^31^P): 0 ppm, rel. delay: 1.0 s, time: 20 h* |
| **3D H(C)P-CCH-TOCSY**  (Marino et al. 1995) | *700 MHz, ns: 144, sw(f3,^1^H): 10 ppm, sw(f2,^13^C): 44 ppm, sw(f1,^31^P): 44 ppm, aq(f3): 73 ms, aq(f2): 7 ms, aq(f1): 12 ms, o1(^1^H): 4.7 ppm, o2(^13^C): 142.5 ppm, o3(^15^N): 153 ppm, rel. delay: 1.0 s, time: 24 h* |
| **2D BEST-TROSY-H(N)CO**  (Solyom et al. 2013) | **G/U-^13^C/^15^N**: 800 MHz^TXO^, ns: 256, sw(f2,^1^H): 21 ppm, sw(f1,^13^C): 25.3 ppm, aq(f2): 67 ms, aq(f1): 20.5 ms, o1(^1^H): 4.7 ppm, o2(^1^NC): 157.53 ppm, o3(^15^N): 153 ppm, rel. delay: 0.3 s, time: 1.5 h  800 MHz^TXO^*, ns: 256, sw(f2,^1^H): 21 ppm, sw(f1,^13^C): 25.3 ppm, aq(f2): 67 ms, aq(f1): 20.5 ms, o1(^1^H): 4.7 ppm, o2(^15^N): 153 ppm, o3(^13^C): 157.5 ppm, rel. delay: 0.3 s, time: 3.5 h* |
| **2D H5(C5C4)N3**  Modified from (Dallmann et al. 2013) | **Optimized for U:**  *950 MHz, ns: 64, sw(f2,^1^H): 10 ppm, sw(f1,^15^N): 101 ppm, aq(f2): 54 ms, aq(f1): 11 ms, o1(^1^H): 4.7 ppm, o2(^15^N): 158.5 ppm, rel. delay: 1.0 s, time: 5 h*  **Optimized for C:**  *950 MHz, ns: 256, sw(f2,^1^H): 10 ppm, sw(f1,^15^N): 101 ppm, aq(f2): 54 ms, aq(f1): 14 ms, o1(^1^H): 4.7 ppm, o2(^15^N): 196 ppm, rel. delay: 1.0 s, time: 22 h* |
| **2D H5(C5)C4**  Modified from (Dallmann et al. 2013) | **G/U-^13^C/^15^N**: 900 MHz, ns: 256, sw(f2,^1^H): 10 ppm, sw(f1,^13^C): 40 ppm, aq(f2): 56 ms, aq(f1): 15.4 ms, o1(^1^H): 4.7 ppm, o2(^13^C): 165.8 ppm, rel. delay: 1.0 s, time: 22 h  *950 MHz, ns: 32, sw(f2,^1^H): 10 ppm, sw(f1,^13^C): 20 ppm, aq(f2): 54 ms, aq(f1): 23 ms, o1(^1^H): 4.7 ppm, o2(^13^C): 165.8 ppm, rel. delay: 1.0 s, time: 2.5 h* |
| **2D H6(C6N1)C2**  (Fürtig et al. 2004) | *600 MHz, ns: 90, sw (f2, ^1^H): 10 ppm, sw(f1,^13^C): 110 ppm, aq(f2): 85 ms, aq(f1): 15 ms, o1(^1^H): 4.7 ppm, o2(^13^C): 155 ppm, rel. delay: 1.0 s, time: 16 h* |

**Supplementary References**

Dallmann A, Simon B, Duszczyk MM, Kooshapur H, Pardi A, Bermel W, Sattler M (2013) Efficient detection of hydrogen bonds in dynamic regions of RNA by sensitivity-optimized NMR pulse sequences. Angew Chem Int Ed Engl 52:10487–10490. https://doi.org/10.1002/anie.201304391

Favier A, Brutscher B (2011) Recovering lost magnetization: polarization enhancement in biomolecular NMR. J Biomol NMR 49:9–15. https://doi.org/10.1007/s10858-010-9461-5

Fiala R, Jiang F, Sklenar V (1998) Sensitivity optimized HCN and HCNCH experiments for 13C/15N labeled oligonucleotides. J Biomol NMR:373–383. https://doi.org/10.1023/A:1008369515755

Fürtig B, Richter C, Bermel W, Schwalbe H (2004) New NMR experiments for RNA nucleobase resonance assignment and chemical shift analysis of an RNA UUCG tetraloop. J Biomol NMR 28:69–79. https://doi.org/10.1023/B:JNMR.0000012863.63522.1f

Kay LE, Xu GY, Singer AU, Muhandiram DR, Forman-Kay JD (1993) A Gradient-Enhanced HCCH-TOCSY Experiment for Recording Side-Chain 1H and 13C Correlations in H2O Samples of Proteins. J Magn Reson B 1993:333–337. https://doi.org/10.1006/jmrb.1993.1053

Marino JP, Schwalbe H, Anklin C, Bermel W, Crothers DM, Griesinger C (1995) Sequential correlation of anomeric ribose protons and intervening phosphorus in RNA oligonucleotides by a 1H, 13C, 31P triple resonance experiment: HCP-CCH-TOCSY. J Biomol NMR 5:87–92. https://doi.org/10.1007/BF00227473

Mori S, Abeygunawardana C, Johnson MO, van Zijl PC (1995) Improved sensitivity of HSQC spectra of exchanging protons at short interscan delays using a new fast HSQC (FHSQC) detection scheme that avoids water saturation. J Magn Reson B 108:94–98. https://doi.org/10.1006/jmrb.1995.1109

Mueller L, Legault P, and Pardi A (1995) Improved RNA structure determination by detection of NOE contacts to Exchange-Broadened amino protons. J Am Chem Soc 1995:11043–11048. https://doi.org/10.1021/ja00150a001

Pardi A, Nikonowicz P (1992) Simple procedure for resonance assignment of the sugar protons in carbon-13 labeled RNAs. J Am Chem Soc 1992:9202–9203. https://doi.org/10.1021/ja00049a070

Piotto M, Saudek V, Sklenár V (1992) Gradient-tailored excitation for single-quantum NMR spectroscopy of aqueous solutions. J Biomol NMR 2:661–665. https://doi.org/10.1007/BF02192855

Richter C, Kovacs H, Buck J, Wacker A, Fürtig B, Bermel W, Schwalbe H (2010) 13C-direct detected NMR experiments for the sequential J-based resonance assignment of RNA oligonucleotides. J Biomol NMR 47:259–269. https://doi.org/10.1007/s10858-010-9429-5

Schanda P, Brutscher B (2005) Very fast two-dimensional NMR spectroscopy for real-time investigation of dynamic events in proteins on the time scale of seconds. J Am Chem Soc 127:8014–8015. https://doi.org/10.1021/ja051306e

Sklenar V, Piotto M, Leppik R, Saudek V (1993) Gradient-Tailored Water Suppression for 1H-15N HSQC Experiments Optimized to Retain Full Sensitivity. Journal of Magnetic Resonance, Series A 1993:241–245. https://doi.org/10.1006/jmra.1993.1098

Solyom Z, Schwarten M, Geist L, Konrat R, Willbold D, Brutscher B (2013) BEST-TROSY experiments for time-efficient sequential resonance assignment of large disordered proteins. J Biomol NMR 55:311–321. https://doi.org/10.1007/s10858-013-9715-0
